# A novel phosphoramide compound, DCZ0805, shows potent anti-myeloma activity via the NF-κB pathway

**DOI:** 10.1186/s12935-021-01973-1

**Published:** 2021-05-30

**Authors:** Xuejie Gao, Bo Li, Anqi Ye, Houcai Wang, Yongsheng Xie, Dandan Yu, Zhijian Xu, Bingqing Shi, Hui Zhang, Qilin Feng, Ke Hu, Yong Zhang, Cheng Huang, Guang Yang, Jumei Shi, Weiliang Zhu

**Affiliations:** 1 Department of Hematology, Shanghai Tenth People’s Hospital, Tongji University School of Medicine, 301 Yanchang Road, Shanghai, 200072 China; 2 CAS Key Laboratory of Receptor Research, Drug Discovery and Design Center, Shanghai Institute of Materia Medica, Chinese Academy of Sciences, 555 Zuchongzhi Road, Shanghai, 201203 China; 3 Shanghai Children’s Medical Center, Shanghai JiaoTong University School of Medicine, Shanghai, People’s Republic of China

**Keywords:** Multiple myeloma, Anti-tumor activity, NF-κB

## Abstract

**Background:**

Multiple myeloma (MM) is a highly aggressive and incurable clonal plasma cell disease with a high rate of recurrence. Thus, the development of new therapies is urgently needed. DCZ0805, a novel compound synthesized from osalmide and pterostilbene, has few observed side effects. In the current study, we intend to investigate the therapeutic effects of DCZ0805 in MM cells and elucidate the molecular mechanism underlying its anti-myeloma activity.

**Methods:**

We used the Cell Counting Kit-8 assay, immunofluorescence staining, cell cycle assessment, apoptosis assay, western blot analysis, dual-luciferase reporter assay and a tumor xenograft mouse model to investigate the effect of DCZ0805 treatment both in vivo and in vitro.

**Results:**

The results showed that DCZ0805 treatment arrested the cell at the G0/G1 phase and suppressed MM cells survival by inducing apoptosis via extrinsic and intrinsic pathways. DCZ0805 suppressed the NF-κB signaling pathway activation, which may have contributed to the inhibition of cell proliferation. DCZ0805 treatment remarkably reduced the tumor burden in the immunocompromised xenograft mouse model, with no obvious toxicity observed.

**Conclusion:**

The findings of this study indicate that DCZ0805 can serve as a novel therapeutic agent for the treatment of MM.

## Introduction

Multiple myeloma (MM) is the second most common malignancy in the hematologic system and is characterized by the latent accumulation of secretory plasma cells in bone marrow [1]. Novel drugs, such as proteasome inhibitors and immunomodulatory drugs (IMiDs), combined with autologous stem cell transplantation (ASCT) paved the way toward a prolonged survival and durable responses [2, 3]. Nevertheless, MM is still incurable and patients are prone to relapse [4]. Therefore, it is of great urgency to identify new targeted drugs for the improved treatment and management of this condition.

Nuclear factor-kappa B (NF-κB) is a DNA-binding transcription factor that can regulate gene transcription by translocating into the cell nucleus and binding specific target sites [5, 6]. The NF-κB signaling pathway plays a crucial part in promoting tumorigenesis, angiogenesis and tumor-microenvironment crosstalk [7–9]. Abnormal and persistent activation of this signaling pathway has been reported in a wide range of malignancies, including MM [10]. Indeed, dysregulation of the NF-κB pathway occurs in MM, contributing to uncontrolled proliferation and resistance to apoptosis [11]. To develop novel anti-myeloma drugs with high efficiency and safety profiles is of great value for improving the remission rate and prolonging the overall survival of patients with MM. Natural compounds and their derivatives, such as arsenic trioxide, resveratrol, and homoharringtonine etc., have been demonstrated significant efficacy in multiple tumors, including hematological malignancies. Our group has long been committed to the research and development of new drugs based on natural compounds. DCZ0805, a newly synthesized compound comprising pterostilbene and osalmide, has been identified as a potential therapeutic agent based on virtual screening of a compound library in our lab. With the purpose of investigating the therapeutic effects of DCZ0805 and elucidate the molecular mechanisms underlying its potential anti-myeloma activity, we have carried out the following work.

## Results

### DCZ0805 exerts potent anti-proliferative activity in MM cell lines

**Fig. 1:**
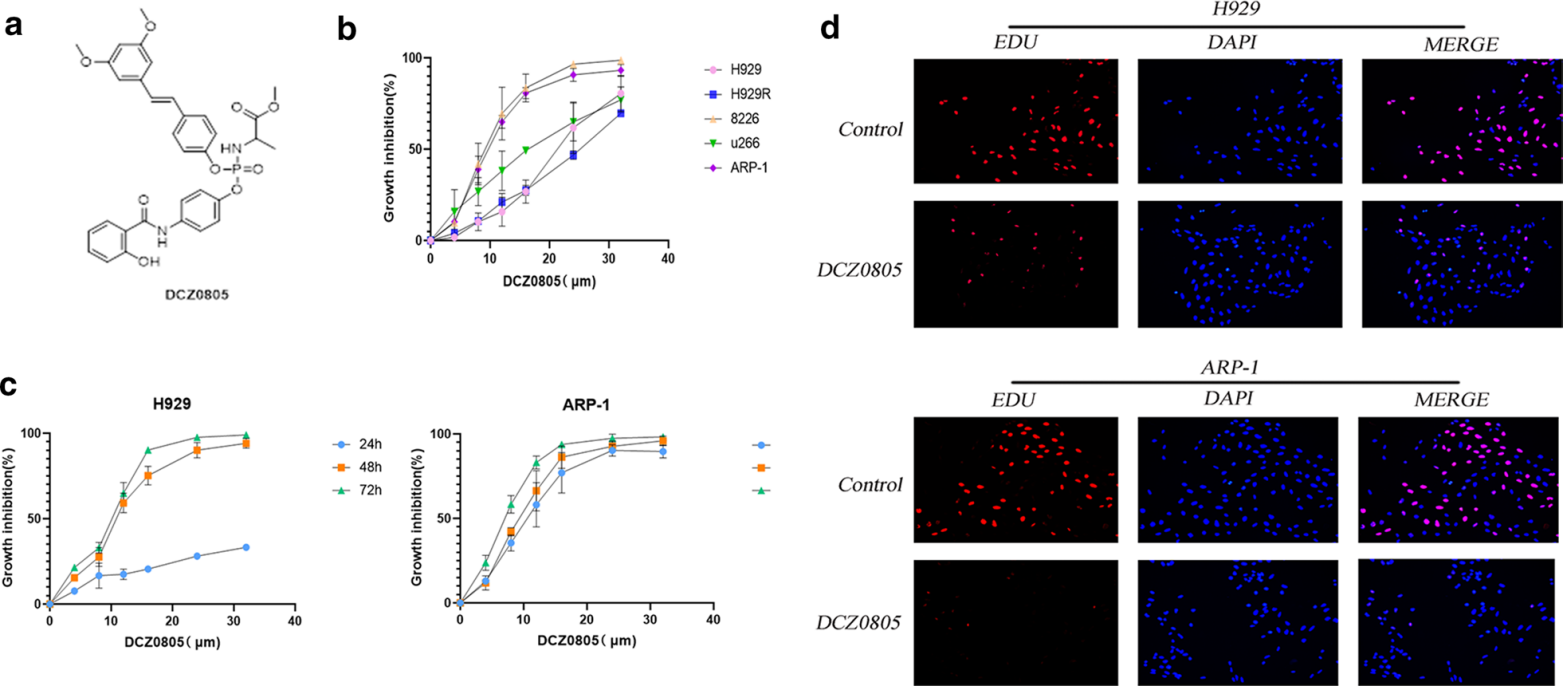
Cytotoxicity of DCZ0805 towards human MM cell lines. **a** Chemical structure of DCZ0805. **b** MM cell lines (H929, ARP-1, U266, RPMI-8226 and H929R) were treated with DCZ0805 (0–32 µM) for 48 h, followed by assessment for cell viability. **c** H929 and ARP-1 cells were treated with DCZ0805 (0–32 µM) and processed at the indicated times, then assessed the cell viability. **d** Micrographs (original magnification, × 50) of proliferation via EdU assay (nuclear staining with DAPI, blue; proliferating cells stained with EdU, red)

DCZ0805, with the molecular weight of 632.6 Da, is a novel compound synthesized by our group (Fig. 1a). In our previous study, we had demonstrated that pterostilbene inhibited the proliferation of MM cells in a concentration- and time-dependent manner [12]. Compared with pterostilbene, the newly synthesized compound, DCZ0805, exhibited a more potent anti-myeloma activity. To investigate whether DCZ0805 has anti-proliferative activity, MM cell lines were treated with various doses of the compound. A CCK-8 assay was applied to evaluate the cell viability after incubation for 48 h. The resulting IC50 (half-maximal inhibitory concentration) values were as follows: 21.02 μM (H929), 11.87 μM (ARP-1), 15.36 μM (U266), 8.73 μM (RPMI-8226), 23.95 μM (H929R) (Fig. 1b). The results showed that DCZ0805 not only exerts its cytotoxic effects in a dose-dependent manner in MM cell lines, but also in a time-dependent manner (Fig. 1c). Moreover, compared with the control group, there were fewer EdU insertions after DCZ0805 treatment (Fig. 1d). These findings together demonstrated that DCZ0805 could effectively inhibited MM cell proliferation.

### DCZ0805 induced G0/G1 phase arrest in MM cells

**Fig. 2:**
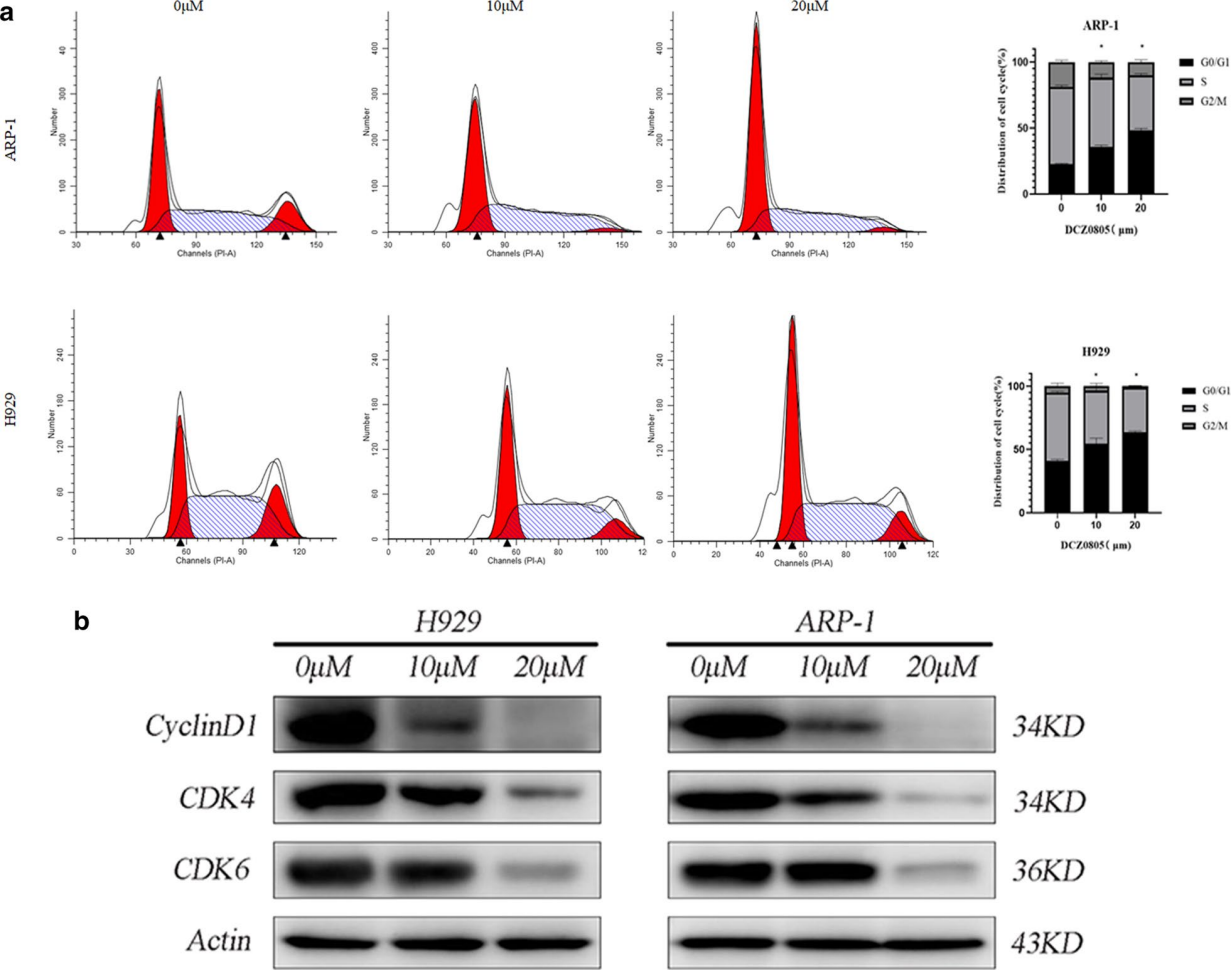
DCZ0805 arrests the cell cycle at G0/G1 phase in MM cells. **a** H929 and ARP-1 cells were treated with DCZ0805 (0, 10 and 20 µM) for 24 h, stained with PI and analyzed by flow cytometry. Bar graphs show the percentage of cells in the G0/G1, S and G2/M phases of the cell cycle. *P < 0.05 compared with the control group (G0/G1 stage). **b** Western blot analysis of the protein expression levels of cyclin D1, CDK4, and CDK6, with β-actin used as an internal reference index

As the induction of cell cycle arrest is a crucial mechanism of action for many anticancer drugs [13]. We used flow cytometry to detect changes in cell cycle progression to explore whether DCZ0805 had an effect on the cell cycle (Fig. 2a). After 24 h of treatment with DCZ0805 (0, 10 and 20 μM), cell cycle progression analysis showed that the proportion of cells in G0/G1 phase increased significantly. Furthermore, consistent with the result of G0/G1 phase arrest, the levels of the G0/G1 phase-related proteins including cyclin D1, CDK6 and CDK4 were significantly reduced in a dose-dependent manner (Fig. 2b). Overall, the above data demonstrated that DCZ0805 may make MM cells stagnant in G0/G1 phase through decreasing the expression of certain key cell cycle regulatory proteins.

### DCZ0805-induced apoptosis activation in MM cells

**Fig. 3:**
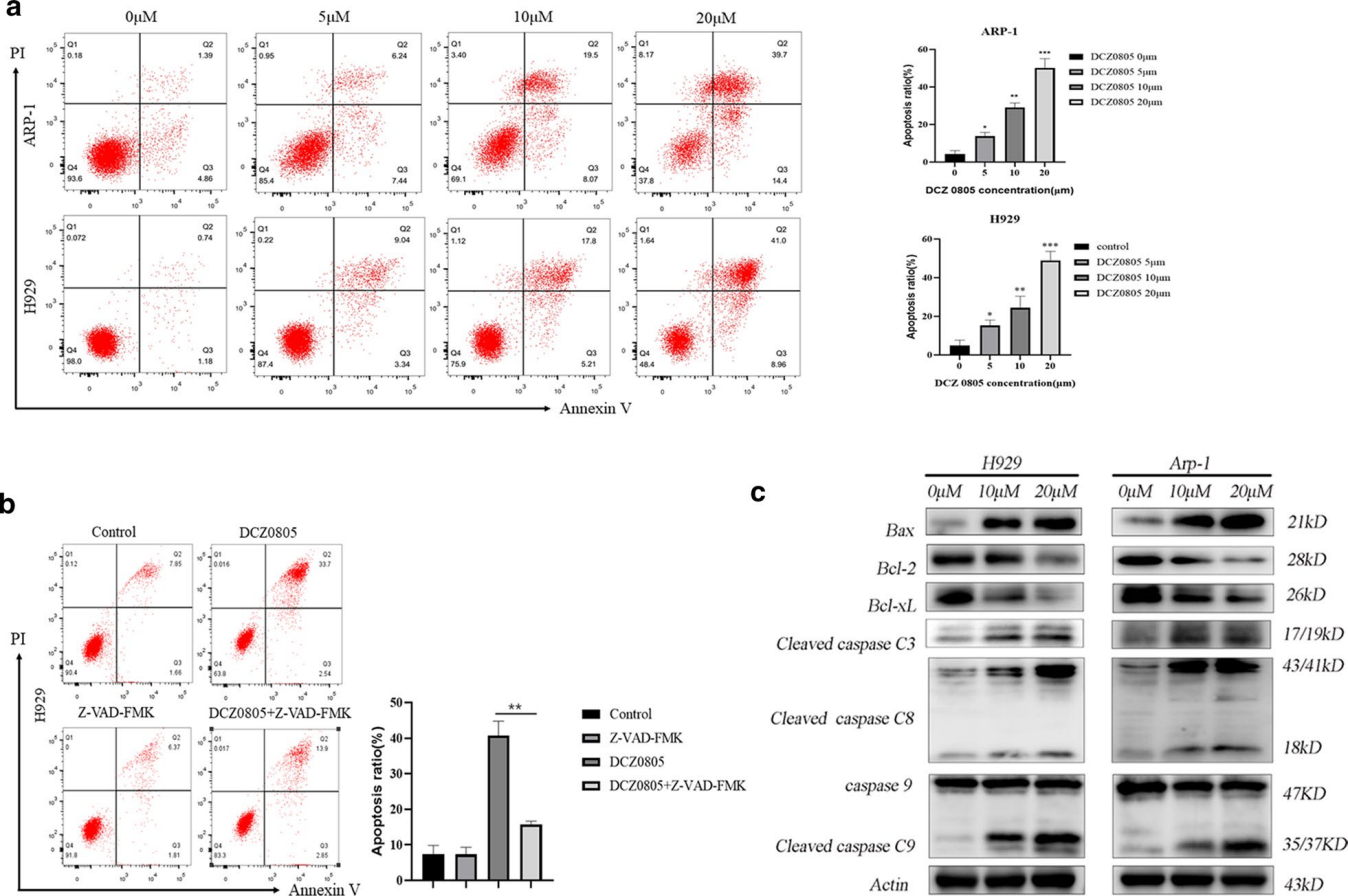
DCZ0805 induces MM cells apoptosis. **a** Cells were treated with DCZ0805 for 48 h and analyzed by FACS using Annexin V/PI staining. Columns show the percentage of Annexin-V positive cells from three independent experiments, data shown as the means ± standard deviation. (*P < 0.05, **P < 0.01, ***P < 0.001 compared with the 0 µM group, or as indicated). **b** Cells were incubated with or without the pan-caspase inhibitor Z-VAD-FMK, and then treated with 15 µM DCZ0805 for 48 h, stained with Annexin V/PI and analyzed by FACS. Data are represented as mean ± SD of three independent experiments and **P < 0.01, by unpaired two-tailed Student's t tests, compared with the DCZ0805-treated group. **c** Detect the expression levels of apoptosis-related proteins by Western blot

It’s known that dysfunctional apoptosis is a fundamental aspect of the tumor biology [14]. To investigate whether MM cell apoptosis is correlated to DCZ0805-induced cytotoxicity, MM cells were treated with different doses of DCZ0805. Flow cytometry plots showed that DCZ0805 induced apoptosis of MM cells in a dose-dependent manner (Fig. 3a). Additionally, as shown in Fig. 3b, the pan-caspase inhibitor, Z-VAD-FMK significantly abrogated DCZ0805-induced apoptosis. This confirmed that DCZ0805-induced cytotoxicity is mediated, at least in part, by caspase-dependent apoptosis. To further shed some light on the potential mechanism underlying apoptosis, apoptosis-related protein expression was examined using western blot analysis. Our results showed that DCZ0805 markedly activated caspase-8 and caspase-3, which are correlated with the extrinsic pathway of apoptosis. Not only that, DCZ0805 reduced the expression of the anti-apoptotic proteins Bcl-2 and Bcl-xL, and up-regulated the expression of the pro-apoptotic protein Bax, all of which are involved in the intrinsic pathway of apoptosis (Fig. 3c). Increased procaspase-9 substrate cleavage was also observed (Fig. 3c). Taken together, DCZ0805 induced MM cells apoptosis via both the intrinsic mitochondrial apoptotic pathway and extrinsic caspase apoptotic pathway.

### Involvement of the NF-κB pathway in DCZ0805-mediated regulation in MM cell lines

**Fig. 4:**
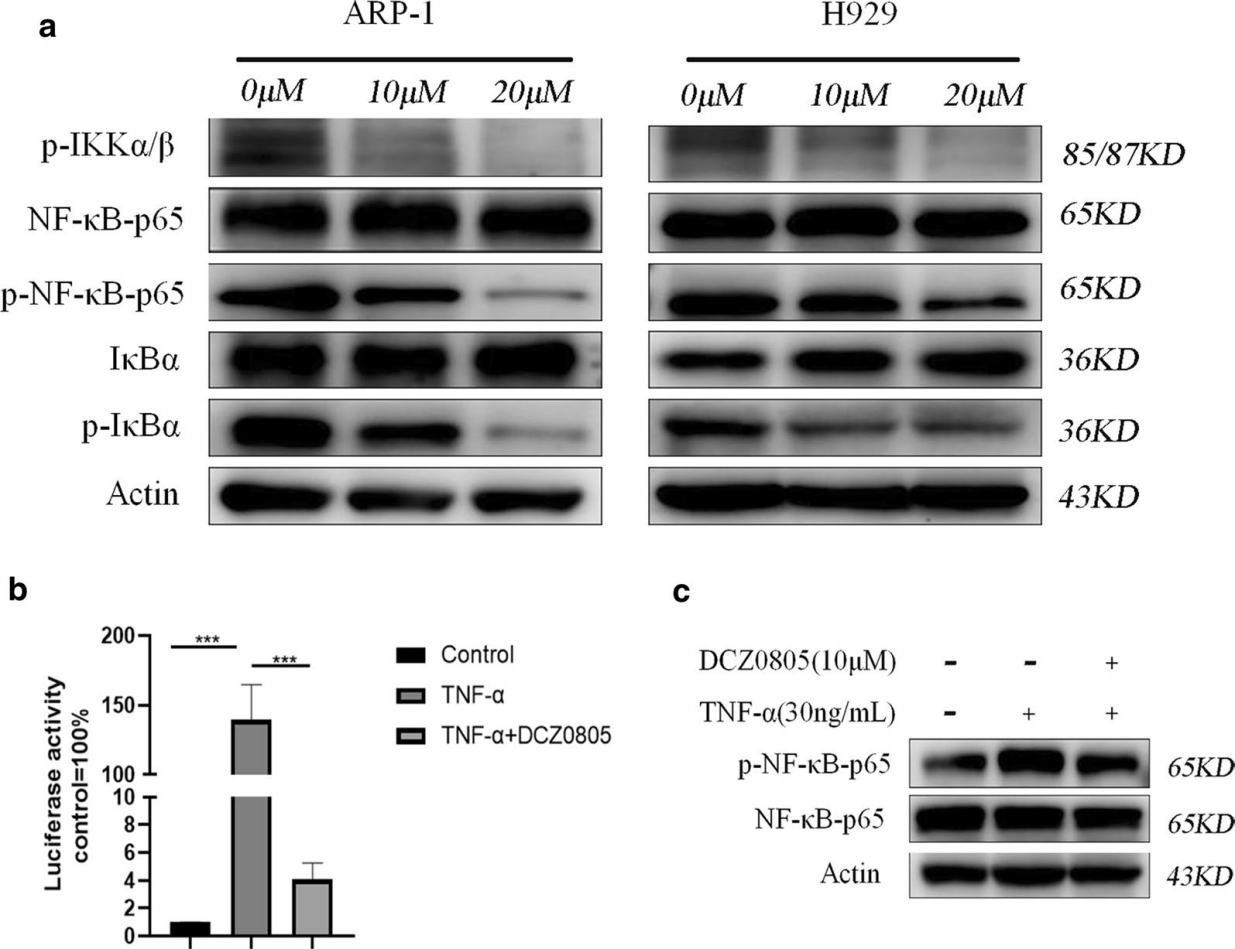
Mechanism of DCZ0805-induced tumor inhibition. **a** H929 and ARP-1 cells were cultured with 10 μM and 20 μM DCZ0805 for 48 h. Western blot analysis was used to evaluate the protein expression level of NF-κB, p-NF-κB, IκBα, p-IκBα, p-IKKα/β and Actin. **b** NF-κB transcriptional activity in ARP-1 cells treated with DCZ0805 (10 μM, 48 h), alone or in combination with TNF-α (30 ng/mL). ***P < 0.001 compared to the control group, data represent the mean ± SD from three separate experiments. **c** Levels of total and phosphorylated NF-κB in ARP-1 cells treated with DCZ0805 (10 μM, 48 h) alone or in combination with TNF-α (30 ng/mL)

Just as NF-κB is highly involved in the occurrence and progression of MM [15], we found that DCZ0805 treatment (0, 10 and 20 μM, for 48 h) patently suppressed the phosphorylation of IκBα and NF-κB-p65, with no obvious impact on total IκBα and NF-κB-p65 protein levels (Fig. 4a). The activated IKK complex is responsible for IκB phosphorylation and degradation, thus contributing to the release and translocation of free NF-κB dimers. The p-IKKα/β was also decreased after DCZ0805 treatment (Fig. 4a). And then, effect of DCZ0805 on transcriptional activity of NF-κB measured by dual-luciferase reporter assay. The results revealed that TNF-α induced significant activation of NF-κB transcription, but the effect was reversed by DCZ0805 in ARP-1 cells (Fig. 4b). We also chose the pathway-specific activator TNF-α to stimulate the NF-κB pathway, the results showed that even in the presence of TNF-α, DCZ0805 could still inhibit the NF-κB pathway (Fig. 4c). Overall, these results showed that DCZ0805 strongly suppressed the NF-κB pathway, which contribute to the anti-proliferative effect of DCZ0805.

### DCZ0805 shows anti-tumor activity in vivo

**Fig. 5:**
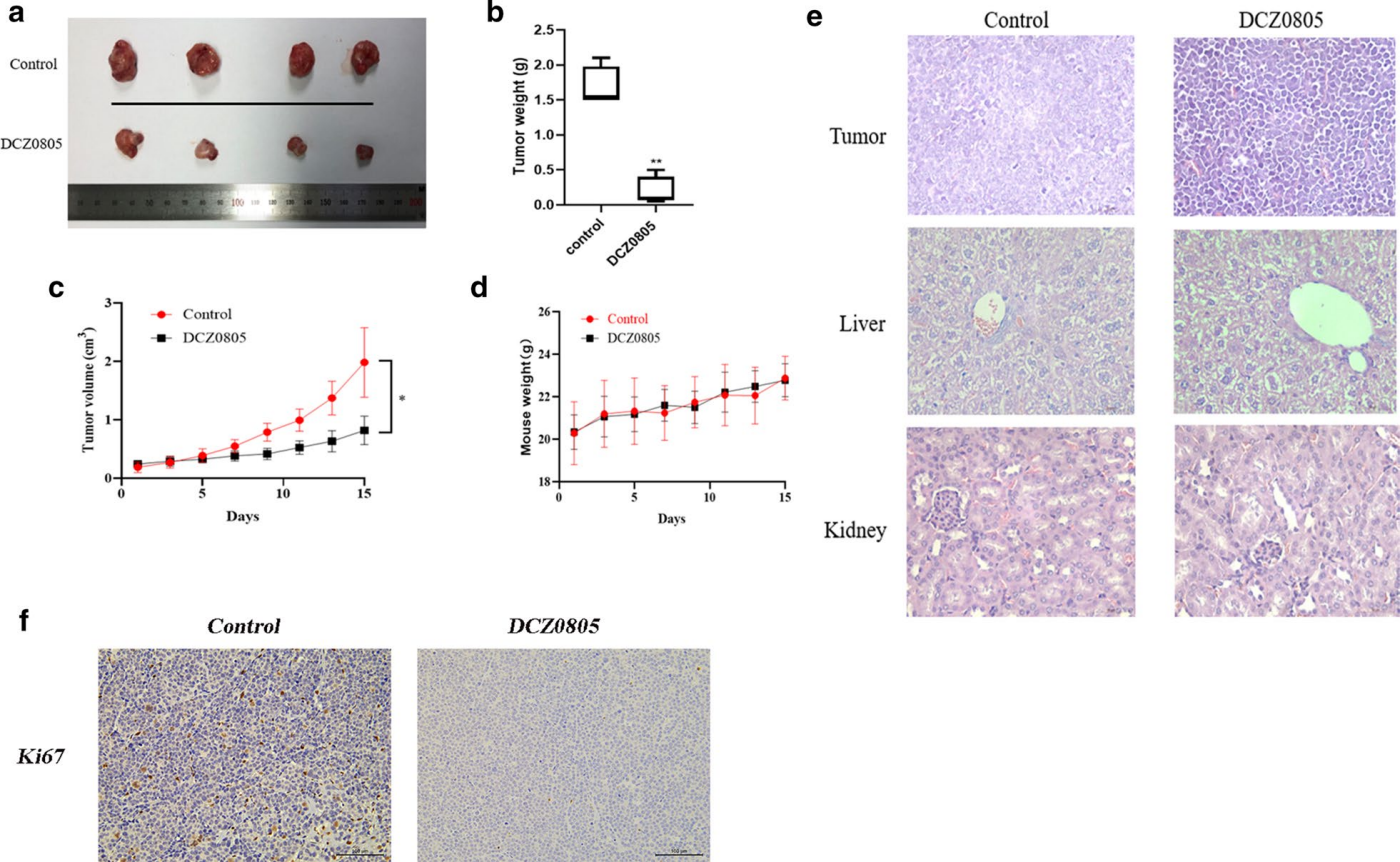
DCZ0805 shows anti-tumor activity in a xenograft mouse model. **a** Photographs of tumor samples collected from MM-xenograft mice treated with DCZ0805 or the vehicle for 15 days. **b** The excised tumors were weighed on the 15th day of treatment. **P < 0.01 compared to the control group. **c** Tumor growth curve of MM-xenograft mice treated with DCZ0805 or vehicle for 15 days. Mean tumor volume (cm^3^) and standard deviation are shown (n = 4 mice/group, *P < 0.05 compared to the vehicle group. **d** The weight of mice was assessed daily for 15 days. Values are expressed as the means ± standard deviation. The difference between groups was not significant (P > 0.05). **e** H&E staining of tumor, liver and kidney sections removed from MM-xenograft mice treated with DCZ0805 or the vehicle; 400 × magnification. **f** Ki67 staining of the control and DCZ0805-treated xenograft tumor tissues

To further investigate the effect of DCZ0805 in vivo, we established a human myeloma xenograft model. We randomly assigned the tumor-bearing mice into the control group or DCZ0805 treatment group. Then each group of mice were intraperitoneally administered vehicle or DCZ0805 (40 mg/kg), respectively. After 15 consecutive days of injection, compared with the control group, the DCZ0805 treatment group showed a clear reduction in tumor size (Fig. 5a–c). In fact, side effects caused by chemotherapy often impair nutritional status including weight loss. Interestingly, no significant difference of the mice weights between the DCZ0805 and control group was observed in the course of treatment (Fig. 5d). Further, histomorphology evaluated by H&E staining showed that the area and degree of tumor necrosis were more severe in DCZ0805-treated mice than in vehicle-treated mice. At the same time, no significant changes in liver and kidney tissues between the two groups (Fig. 5e). Moreover, a decrease in Ki-67 expression was observed in the DCZ0805-treated group (Fig. 5f), further confirming that DCZ0805 has potential anti-proliferative effect in vivo. These findings indicate that DCZ0805 is a promising drug for MM treatment, and the side effects were well-tolerated in xenograft mice.

## Discussion

Over the past few decades, immunomodulatory drugs (thalidomide and lenalidomide) and proteasome inhibitors (bortezomib) have greatly prolonged the survival of MM patients [16]. However, as the disease remains incurable and patients are often subject to relapse, there is a clear need for novel therapeutic strategies targeting defined pathogenetic events in MM. Because of the low cytotoxicity induced in healthy cells, the broad therapeutic spectrum, and high-efficiency proliferation inhibition, natural compounds and their derivatives are being widely studied as antitumor drugs. In the current study, we synthesized a novel derivative of osalmid and pterostilbene, DCZ0805. Through a series of in vitro experiments, it showed a concentration-dependent proliferation inhibitory effect on MM cell lines. To our knowledge, the present study is the first to demonstrate that DCZ0805 could inhibit MM cells by suppressing the NF-κB pathway. In addition, with the prolongation of the treatment time, the inhibitory effect on ARP-1 and H929 cells became more apparent.

Molecularly, the cell cycle, which involves a series of tightly integrated events, is regulated by protein kinase complexes, each of which consists of a CDK and a cyclin [17]. CDK activity requires the binding of cyclins, and cyclin D1/CDK4/CDK6 complexes play a key role in controlling cell progression from the G1 to the S phase [17, 18, 22]. In this study, treatment of MM cells with DCZ0805 can down-regulate the expression levels of the above-mentioned cell cycle-related proteins, eventually arrested the MM cells in the G0/G1 stage.

Impaired apoptosis leads to oncogenesis and progression of most malignant tumors, so targeted regulation of apoptosis pathway may improve the efficacy of cancer therapies and mitigate resistance [19]. Apoptosis signaling pathways mainly include the mitochondria-mediated pathway (intrinsic) and the death receptor pathway (extrinsic) [20]. Bcl-2 family members, the core regulators of the intrinsic pathway, regulate the balance between pro-apoptotic (Bax) and anti-apoptotic (Bcl-2) proteins, which determines whether intrinsic apoptosis would be initiated. In the extrinsic pathway, caspase-3 can be cleaved by the activated initiator caspase-8 and caspase 9, ultimately resulting in apoptosis [21, 22]. In the current study, DCZ0805 activated both external and internal apoptotic pathways through the activation of caspase-8, -3, -9 and the suppression of Bcl-2 and Bcl-xL proteins. Of note, flow cytometry analysis showed that as the dose increased, the proportion of apoptotic cells also increased, and this finding was confirmed by a rescue experiment with the use of caspase inhibitor Z-VAD-FMK.

NF-κB is a dimeric transcription factor belonging to a family of proteins with a common conserved DNA-binding region. NF-κB complexes typically exist in an inactive state mainly through interactions with the inhibitor of κB (IκB) family proteins in the cytoplasm [23]. However, once the IκB protein is phosphorylated by the IKK complex, it goes through rapid ubiquitination and degradation, which culminates in the release of the NF-κB complexes from their inhibitory interaction. Then, the NF-κB dimers translocate into the nucleus, where they bind to specific DNA sites and regulate the expression of genes involved in the cell proliferation control and the cell survival regulation [24].

A large amount of data indicate that the NF-κB pathway is widely involved in mediating the initiation and progression of human tumors, including of course MM [25]. The NF-κB pathway plays a key role in promoting the growth of cancer, thereby affecting the survival and prognosis of patients with MM. Interestingly, NF-κB can directly regulate a potent anti-apoptotic response through inhibiting the expression of anti-apoptotic Bcl-2 and Bcl-xL genes [26]. Moreover, the nuclear translocation of NF-κB could coincide with the G0/G1 phase of the cell cycle, which can mediate the expression of cyclin D1 [27]. In the present study, after DCZ0805 treatment, phosphorylated IκBα and NF-κB proteins were dramatically decreased in MM cells. Consistent with these findings, due to the suppression of NF-κB activity, the expression levels of above-mentioned NF-κB targets, including Bcl-2, Bcl-xL and cyclin D1, were also decreased. In addition, it is well known that TNF-α induces activation of the NF-κB pathway. However, we found that even under the stimulation of the specific activator TNF-α, DCZ0805 could still significantly inhibited the activation of the NF-κB pathway. We further demonstrated the inhibitory effects of DCZ0805 on NF-κB pathway through the inhibition of NF-κB transcriptional activity. Taken together, it can be inferred that DCZ0805 inhibited the growth and proliferation of MM cells through inactivation of the NF-κB pathway.

In view of the finding that DCZ0805 exhibits cytotoxicity and induces apoptosis towards MM cells in vitro, a tumor transplantation model of nude mice was established to observe its effect in vivo. Mice were randomly divided into control and DCZ0805 treatment groups. Intraperitoneal administration of 40 mg/kg DCZ0805 inhibited tumor growth significantly. At the same time, no indications of unexpected toxicity or toxicity-related deaths were recorded. Furthermore, there was no significant differences in the weights of mice from the two groups was observed.

Overall, these results support the potential clinical application of DCZ0805 as a novel anti-tumor agent for MM. However, to fully utilize this compound as an effective therapeutic tool, further research is needed to clarify the exact cellular mechanisms underlying its activity.

## Materials and methods

### Cell lines and cell culture

Human MM cell lines H929, ARP-1, U266 and RPMI-8226 were purchased from the American Type Culture Collection (ATCC, Manassas, USA). Bortezomib-resistant MM cell line H929R was provided by Jian Hou (Department of Hematology, Changzheng Hospital, The Second Military Medical University, Shanghai, China). According to Professor Hou’s previous report, H929R cell line was obtained by stepwise increasing extracellular concentrations of bortezomib over a period of 8 months. MM cell lines were cultured in RPMI-1640 medium containing 10% fetal bovine serum (FBS, Sigma), 100 IU/ml penicillin, and 100 μg/ml streptomycin (GIBCO, Grand Island, USA). All cells were maintained at 37 °C in 5% carbon-dioxide, and culture medium was changed every other day.

### Reagents and antibodies

DCZ0805 was synthesized by the Shanghai Institute of Materia Medica (Chinese Academy of Sciences, Shanghai, China). It was dissolved in dimethyl-sulfoxide (DMSO; Sigma-Aldrich, St. Louis, MO, USA) at a concentration of 100 mM, stored at − 20 °C and diluted at desired concentrations into each well plate with cell suspension. The Cell Counting Kit-8 (CCK-8) was obtained from Yeasen (Shanghai, China), the Annexin-V/propidium iodide (PI) apoptosis detection kit was purchased from BD Pharmingen (Franklin Lakes, USA), and Z-VAD-FMK was obtained from Selleck Chemicals (Houston, USA). Recombinant human TNF-α was obtained from R&D Systems (Minneapolis, MN, USA). Antibodies against cleaved caspase-8, caspase-3, and β-actin were purchased from Cell Signaling Technology (Beverly, USA). Antibodies against CDK4, CDK6, cyclin D1, Bcl-2, Bcl-xL, Bax, caspase-9, IκBα, p-IκBα, p-IKKα/β, NF-κB-p65 and p-NF-κB-p65 were obtained from Abcam (Cambridge, MA, USA).

### Cell viability assay

H929 and ARP-1 cells were seeded in 96-well plates (100 μl/well) at a density of 2 × 10^5^ cells/ml and were treated with 4, 8, 12, 16, 24 and 32 µM DCZ0805 for 24, 48 and 72 h. After incubation at 37 °C and 5% carbon-dioxide, 10 µl CCK-8 solution was added to each well and the cells were incubated for an additional 2 h at 37 °C. Then we measured absorbance at 450 nm using a microplate reader. Data were analyzed using the CalcuSyn (Biosoft, Ferguson, MO), which calculates the half-maximal inhibitory concentration (IC50) for each drug.

### Apoptosis measurement

MM cells are cultured in a 24-well plate at a density of 2 × 10^5^ cells/ml, then different concentrations of DCZ0805 (0, 10, 20 μM) were added to each well for 48 h. Besides, after pre-stimulating the cells with the pan-apoptosis inhibitor Z-VAD-FMK (50 μM) for 2 h, DCZ0805 was added to each well and incubated for 48 h. Cell suspension is collected and supernatant is decanted after centrifugation at 142*g* for 5 min. Then apoptosis was measured using an FITC Annexin V Apoptosis Detection kit (BD Biosciences, Franklin Lakes, NJ, USA). Annexin V fluorescein isothiocyanate (FITC) was used for cell staining at 4 °C for 30 min in the dark, followed by propidium iodide (PI) staining for 10 min. And the BD FASC Canto II flow cytometry (BD Biosciences, San Jose, CA, USA) was used for analysis. Annexin V + /PI− (early apoptosis) and Annexin V + /PI + (late apoptosis) were identified as apoptotic cells.

### Cell cycle analysis

Expose MM cells to different doses of DCZ0805 for 24 h, H929 and ARP-1 cells at a density of 2 × 10^5^ cells/ml were washed with cold PBS, and fixed in 75% ice‑cold ethanol overnight at − 20 °C. The ethanol‑fixed samples were washed with PBS and incubated in 300 µl PI/RNase staining buffer (BD Biosciences) for 15 min at room temperature in the dark. The percentages of cell population that were in each phase of the cell cycle (G0/G1, S, G2/M) were analyzed by ModFit LT 3.2 (Verity Software House, Inc., Topsham, ME, USA).

### 5-Ethynyl-2'-deoxyuridine (EdU) labeling and immunofluorescence

H929 and ARP-1 cells were plated into a 6-well plate at a density of 4 × 10^5^ cells/well, and were incubated with or without 10 µM DCZ0805 at 37 °C for 48 h. Subsequently, cells were incubated with 50 µM EdU (Guangzhou RiboBio Co., Ltd., Guangzhou, China) at 37 °C for 1 h. Then the cells were fixed with 4% paraformaldehyde for 30 min and treated with 0.5% Triton x-100 for 10 min at room temperature. Then the cells were washed 3 times with PBS. Thereafter, the cells were exposed to 100 µl 1X Apollo reaction cocktail (deionized water, apollo^®^ reaction buffer-reagent B, apollo^®^ catalytic buffer -reagent C, apollo^®^ fluorescent dye solution buffer-reagent D, apollo^®^ buffer additives-reagent E) at 37 °C for 30 min and incubated with DAPI at room temperature to stain the cell nuclei for 5 min. A confocal laser-scanning microscope was used to detect stained cells.

### Western blot

H929 and ARP-1 cells were treated with different doses of DCZ0805 for 48 h, then were lysed on ice for 30 min in the lysate buffer (100 mM Tris–HCl, pH 6.8, 4% SDS, 20% glycerol) and the supernatant was collected. BCA method (Beyotime Institute of Biotechnology, Haimen, China) was used for detecting the protein concentration. Samples containing 30 µg protein were isolated by 8–12.5% sodium dodecyl sulfate–polyacrylamide gels [28]. Then different molecular weight proteins were transferred to the nitrocellulose membrane and blocked with 5% non-fat dried milk at room temperature for 1 h. After incubation for 5 h at 4 °C with the primary antibody, the membranes were washed three times with PBST (1 × PBS-0.01% tween-20) for 10 min each. Primary antibodies were as follow: anti-cleaved caspase-8, anti-caspase-3, and anti-β-actin were purchased from Cell Signaling Technology (Beverly, USA); anti-CDK4, anti-CDK6, anti-cyclin D1, anti-Bcl-2, anti-Bcl-xL, anti-Bax, anti-caspase-9, anti-IκBα, anti-p-IκBα, anti-p-IKKα/β, anti-NF-κB-p65 and anti-p-NF-κB-p65 were obtained from Abcam (Cambridge, MA, USA). The membrane was then probed with the corresponding secondary antibody (anti-rabbit or anti-mouse IgG) for 60 min at room temperature and washed three times with PBST. Protein bands were detected using the Odyssey two-color infrared laser imaging system (LI-COR Biosciences, Lincoln, USA).

### Dual-luciferase reporter assay

ARP-1 cells (2 × 10^5^ cells/mL) co-transfected with the p-p65-TA-luc (Beyotime Biotechnology) and Renilla luciferase pTKRL (Promega) plasmids at a 10:1 ratio were treated with DCZ0805 (10 μM, 48 h), with or without TNF-α stimulation (30 ng/mL). Lysates were analyzed using a dual-luciferase assay kit (Promega) according to the manufacturer's protocol.

### Tumor xenograft model

Five-week-old male BALB/C nude mice were purchased from the Shanghai Animal Experimental Center. The mice survived in an air-conditioned room at 24 °C, humidity of 45%, and with 12 h light/dark cycle. The autoclaved food and water were given to mice without limitation and animal experiments were carried out after all mice had been acclimated for at least a week. Human H929 cells (3 × 10^6^) in 100 μL serum-free culture medium were subcutaneously injected into the upper flank region of the nude mice. When the tumor was measurable, mice were randomly assigned to the vehicle group and the DCZ0805 group. The control group was given 100 μL of placebo (10% DMSO, 30% HS-15 and 60% normal saline), while the DCZ0805 group was intraperitoneally injected DCZ0805 with 40 mg/kg [29]. The size of the tumor and the weight of mice are measured daily and the volume of the tumor is calculated as (length × width^2^) × 0.5. At the end of the treatment, all mice were sacrificed by cervical dislocation. The tumor of the mouse was completely stripped from its skin and the vital organs such as liver and kidney were removed and then fixed with 4% paraformaldehyde, followed by H&E and immunohistochemistry (IHC) staining. All operations associated with animal experiments were approved by the Animal Care and Use Committee of Tongji University (Shanghai, China) and the institutional review board of the Shanghai Tenth People’s Hospital (ID: SYXK 2018–0034).

## Statistical analysis

Data are expressed as the means ± standard deviation. Statistical analysis was conducted using an unpaired Student's t-test or one-way analysis of variance followed by least significant difference test for multiple comparisons. All statistical analyses were performed using SPSS version 20.0 statistical analysis software (IBM Corp., Armonk, NY, USA). P < 0.05 was considered statistically significant difference.

## Data Availability

All data generated or analysed during this study are included in this published article.

## Abbreviations

Annexin V-FITC: Fluoresceinated annexin V
ASCT: Autologous stem cell transplantation
Bax: Bcl-2-associated X protein
Bcl-2: B cell lymphoma 2
Bcl-xL: B cell lymphoma extra-large
CDK: Cyclin-dependent kinase
DMSO: Dimethyl sulfoxide
EdU: 5-Ethynyl-2′-deoxyuridine
H&E: Hematoxylin and eosin
IC50: Half-maximal inhibitory concentration
IκB: Inhibitor of κB
IMiDs: Immunomodulatory drugs
MM: Multiple myeloma
NF-κB: Nuclear factor-kappa B
PI: Propidium iodide
